# Longitudinal analysis of new multiple sclerosis lesions with magnetization transfer and diffusion tensor imaging

**DOI:** 10.1007/s00330-023-10173-6

**Published:** 2023-09-02

**Authors:** Monika Gloor, Michaela Andelova, Laura Gaetano, Athina Papadopoulou, Federico Burguet Villena, Till Sprenger, Ernst-Wilhelm Radue, Ludwig Kappos, Oliver Bieri, Meritxell Garcia

**Affiliations:** 1 Division of Radiological Physics, Department of Radiology, University Hospital Basel, Basel, Switzerland; 2 Department of Neurology, University Hospital Basel, Basel, Switzerland; 3 Department of Neurology and Center of Clinical Neuroscience, First Faculty of Medicine, Charles University and General University Hospital in Prague, Prague, Czech Republic; 4 Medical Image Analysis Center (MIAC) AG, Basel, Switzerland; 5 Novartis Institutes for BioMedical Research Basel, Basel, Switzerland; 6 Department of Clinical Research, Faculty of Medicine, University Hospital Basel, Basel, Switzerland; 7 University Hospital Zürich, Zurich, Switzerland; 8 Department of Biomedical Engineering, University of Basel, Allschwil, Switzerland; 9 Division of Neuroradiology, Department of Radiology, University Hospital Basel, Basel, Switzerland; 10 Department of Neuroradiology, University Hospital Zürich, Frauenklinikstrasse 10, 8091 Zurich, Switzerland

**Keywords:** Multiple sclerosis, Balanced steady-state free precession, Quantitative magnetization transfer, Magnetization transfer ratio, Diffusion tensor imaging

## Abstract

**Objective:**

The potential of magnetization transfer imaging (MTI) and diffusion tensor imaging (DTI) for the detection and evolution of new multiple sclerosis (MS) lesions was analyzed.

**Methods:**

Nineteen patients with MS obtained conventional MRI, MTI, and DTI examinations bimonthly for 12 months and again after 24 months at 1.5 T MRI. MTI was acquired with balanced steady-state free precession (bSSFP) in 10 min (1.3 mm^3^ isotropic resolution) yielding both magnetization transfer ratio (MTR) and quantitative magnetization transfer (qMT) parameters (pool size ratio (F), exchange rate (kf), and relaxation times (T1/T2)). DTI provided fractional anisotropy (FA), mean diffusivity (MD), axial diffusivity (AD), and radial diffusivity (RD).

**Results:**

At the time of their appearance on MRI, the 21 newly detected MS lesions showed significantly reduced MTR/F/kf and prolonged T1/T2 parameters, as well as significantly reduced FA and increased AD/MD/RD. Significant differences were already observed for MTR 4 months and for qMT parameters 2 months prior to lesions’ detection on MRI. DTI did not show any significant pre-lesional differences. Slightly reversed trends were observed for most lesions up to 8 months after their detection for qMT and less pronounced for MTR and three diffusion parameters, while appearing unchanged on MRI.

**Conclusions:**

MTI provides more information than DTI in MS lesions and detects tissue changes 2 to 4 months prior to their appearance on MRI. After lesions’ detection, qMT parameter changes promise to be more sensitive than MTR for the lesions’ evolutional assessment. Overall, bSSFP-based MTI adumbrates to be more sensitive than MRI and DTI for the early detection and follow-up assessment of MS lesions.

**Clinical relevance statement:**

When additionally acquired in routine MRI, fast bSSFP-based MTI can complement the MRI/DTI longitudinal lesion assessment by detecting MS lesions 2–4 months earlier than with MRI, which could implicate earlier clinical decisions and better follow-up/treatment assessment in MS patients.

**Key Points:**

*• Magnetization transfer imaging provides more information than DTI in multiple sclerosis lesions and can detect tissue changes 2 to 4 months prior to their appearance on MRI.*

*• After lesions’ detection, quantitative magnetization transfer changes are more pronounced than magnetization transfer ratio changes and therefore promise to be more sensitive for the lesions’ evolutional assessment.*

*• Balanced steady-state free precession–based magnetization transfer imaging is more sensitive than MRI and DTI for the early detection and follow-up assessment of multiple sclerosis lesions.*

## Introduction

Advanced MRI [1, 2], e.g., diffusion-weighted imaging (DWI) [3], diffusion tensor imaging (DTI) [1, 4, 5], and magnetization transfer imaging (MTI) [6–8], are complementary to conventional MRI (cMRI) [1, 2] for tissue characterization in multiple sclerosis (MS).

Information about protons bound to macromolecules, “invisible” on cMRI, is obtained via magnetization exchange between “bound” and “free” protons. The simplest method to yield evidence from the bound protons is by magnetization transfer ratio (MTR) assessment [9–11], a semi-quantitative parameter depending on various sequence parameters and comprising mixed information from various quantitative MT (qMT) parameters. Information from qMT imaging, including the relative amount of the restricted protons (F), exchange rate between the free and restricted proton pools (kf), and longitudinal and transverse relaxation times of the free pool (T1, T2), requires a complex data analysis and longer acquisition time (TA) [6–8, 12, 13].

Diffusion imaging assesses movement of water molecules [4]. DTI provides more information due to a high number of diffusion-weighted images. DTI parameters include the fractional anisotropy (FA), mean diffusivity (MD), axial diffusivity (AD), and radial diffusivity (RD) [4].

Previous studies assessing new MS lesions with MTI/DTI show inconsistent results. The normal-appearing white matter (NAWM) and dirty white matter (DWM) in MS patients differ from normal white matter (NWM) in healthy subjects [10–14]. Magnetization transfer (MT) parameters have been claimed to be more altered in the pre-lesional NAWM than in the NAWM in which no lesions will develop [10, 11, 15], and MS lesions seem not to be associated with the DWM in which no lesions seem to arise [12, 13].

This study investigates the efficacy of DTI and high-resolution balanced steady-state free precession (bSSFP)–based MTI assessing both MTR and qMT parameters longitudinally, with emphasis on the appearance and evolution of new MS lesions.

Contrary to most previous publications, the presented MT-bSSFP technique, in combination with its inherently higher MT sensitivity and higher signal-to-noise ratio (SNR), enables assessment of both MTR and qMTI parameters in an TA of less than 10 min [16, 17].

## Materials and methods

### Patients

Twenty-two MS patients (age range 21–54 years) under disease-modifying therapy (MDT; Table 1), with (sub-)clinically active relapsing–remitting MS (RRMS) or secondary progressive MS (SPMS) with ongoing relapses, independently of the respective EDSS scores, were recruited. Clinical activity was defined as “at least two relapses in the last two years (with at least one relapse in the previous twelve months) or one relapse of cerebral origin as indicated by an MRI performed immediately before the first/baseline-MRI.” Subclinical activity was defined as “evidence of at least one new or markedly enlarging T2w lesion or at least one contrast-enhancing (CE) lesion within one year before the first/baseline-MRI.”

**Table 1:** Overview of the disease duration and the course of the disease-modifying therapy (DMT) of each individual patient. Please note that patient 22 was not under DMT neither at the timepoint of inclusion nor at the end of the study, however for some time during the study

Pt	Sex	Disease duration (years	DMT at baseline	DMT change during the study	DMT at the end of the study
3	M	17	Interferon-beta-1a i.m	Yes	Fingolimod
4	M	8	Natalizumab	Yes	Fingolimod
5	F	16	Interferon-beta-1b s.c	No	Interferon-beta-1b s.c
6	F	10	Fingolimod	No	Fingolimod
7	F	9	Fingolimod	No	Fingolimod
8	M	19	Rituximab	No	Rituximab
9	F	25	Fingolimod	No	Fingolimod
10	M	4	Fingolimod	No	Fingolimod
11	F	5	Glatiramer acetate	No	Glatiramer acetate
12	F	21	Interferon-beta-1a i.m	Yes	Natalizumab
13	F	10	Glatiramer acetate	No	Glatiramer acetate
14	F	29	Glatiramer acetate	No	Glatiramer acetate
15	F	1	Natalizumab	Yes	Fingolimod
16	F	22	Fingolimod	Yes	Rituximab
17	F	6	Natalizumab	No	Natalizumab
18	F	8	Fingolimod	No	Fingolimod
19	M	5	Fingolimod	No	Fingolimod
20	F	3	Interferon-beta-1b s.c.	Yes	Dymethyl fumarate
22	F	7	None	Yes, dimethyl fumarate and teriflunomide during the study	None

Considering three patient dropouts, 19 patients (five males, 14 females, mean age 39.2 years, SD 9.9 years) completed the study.

The study protocol was reviewed and approved by the Institutional Review Board. Written consent was obtained from all patients.

### Magnetic resonance imaging

Patients received eight MRI examinations during the study. For 1 year, they were scanned every 2 months with seven MRI scans (months 0 (baseline), 2, 4, 6, 8, 10, and 12). After a following 1-year break, a last MRI was performed 24 months after baseline MRI (month 24).

MRI examinations were performed at 1.5 T (Avanto, Siemens) with cMRI, MTI, and DTI. Conventional MRI included (1) transversal proton density-weighted (PDw)/T2-weighted (T2w) imaging: TR 5300 ms, TE 24/96 ms, voxel size 1.1 × 0.9 × 3.0 mm; (2) 3D-fluid attenuated inversion recovery (FLAIR) imaging: TR/TE 6000/352 ms, voxel size 1.0 × 1.0 × 1.0 mm^3^; (3) sagittal T2w imaging: TR/TE 3400/331 ms, voxel size 1.0 × 1.0 × 1.0 mm^3^; and (4) 3D-T1w (T1-weighted) magnetization-prepared rapid gradient-echo (MPRAGE) imaging ± contrast: TR/TE 2700/5.03 ms, voxel size 1.0 × 1.0 × 1.0 mm^3^.

MTI (MTR/qMT) comprised a B1 map with a flip angle of 30°, two radiofrequency (RF) spoiled gradient-echo (SPGR) sequences with variable flip angles (3°/17°) for T1 determination, two bSSFP sequences with variable flip angles (15°/35°) for T2 determination, five bSSFP scans with variable RF pulse durations (0.12–1.5 ms) at a flip angle of 35° (TR 2.55–3.93 ms), and five bSSFP scans with variable flip angles (5°–35°) and constant RF pulse duration (0.12 ms). MT-bSSFP imaging was acquired within 10 min (144 slices, in-plane resolution 192, voxel size of 1.3 × 1.3 × 1.3 mm^3^).

For DTI, 30 non-collinear diffusion-weighting gradients were used (*b* = 900 s/mm^2^, 10 *b* = 0 acquisitions, 2 averages, 55 slices, in-plane resolution 128, voxel size 2 × 2 × 2 mm^3^, TE/TR 95/8900 ms, TA 10.5 min).

Within the first study year, there was a total of six missed MRIs by four patients and in six patients the DTI examination was not performed at one timepoint.

### Data processing

Softwares FSL [18] and AFNI [19] were used for brain extraction and registration of all images (cMRI/MTI/DTI and lesions’ masks) to one bSSFP image of the first MR examination. Effective flip angles were calculated on a pixel-by-pixel base after B1 image registration. T1/T2 relaxation times of the free proton pool were calculated with DESPOT1/DESPOT2 [20, 21]. The two-pool model parameters F and kf were estimated from a pixel-wise nonlinear least-squares fit to the bSSFP images [13]. MTR maps were calculated from an MT-weighted and a non-MT-weighted bSSFP scan [9].

3D parameter maps for T1/T2/F/kf and MTR were extracted for each examination timepoint.

Diffusion-weighted images were motion-corrected, the diffusion tensor was fitted using FSL [18], and acquired DWI maps (FA/MD/AD/RD) were aligned to one bSSFP image of the first MR examination.

For lesions’ detection, PDw/T2w and 3D-FLAIR sequences were revised. For initial co-registration, all newly detected MS lesions were manually drawn as masks on PDw images with the software tool ITK-SNAP [22] by a resident in neurology and verified/corrected by an experienced consultant neuroradiologist. For every new lesion, a corresponding reference mask of approximately the same size was drawn in the contralateral NAWM. All new lesion masks were drawn slightly smaller than the visible lesions’ size to avoid partial volume contamination from adjacent tissue and guarantee a safe lesion margin. Very small lesions and lesions not clearly separable from preexisting lesions were not considered.

**Fig. 1:**
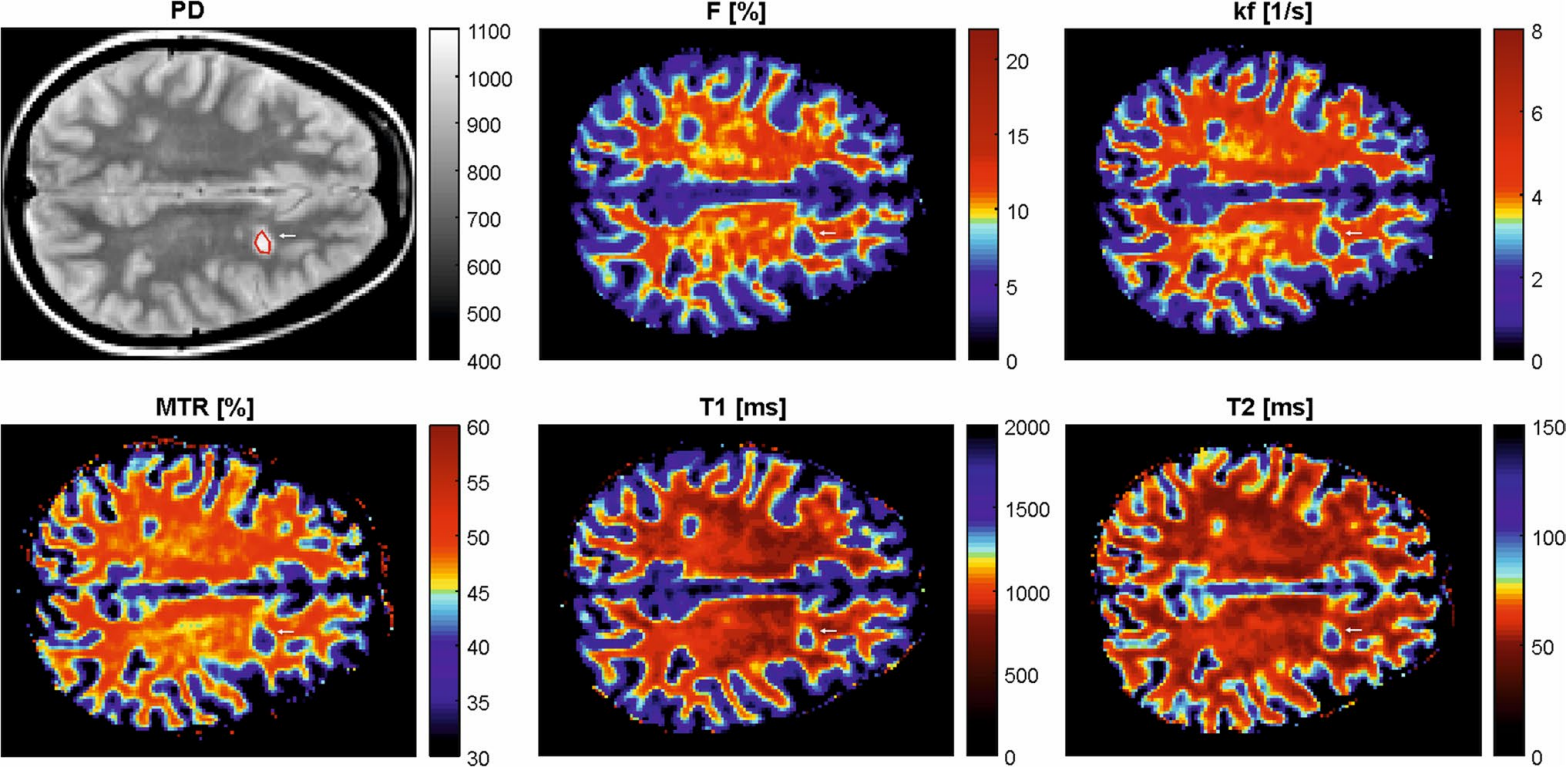
MT parameter maps. An exemplary MS lesion was manually segmented on a PD-weighted image, marked by an arrow. The lesion mask was superimposed onto the individual MT parameter maps to derive median values for each lesion at every timepoint

**Fig. 2:**
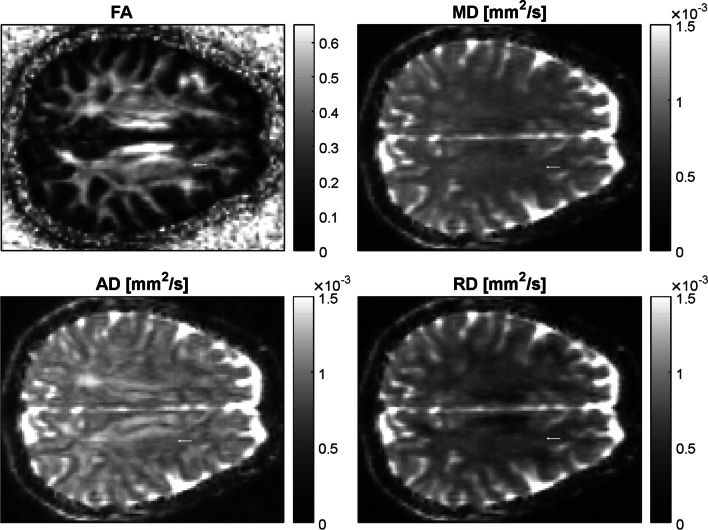
DTI maps. Exemplary DTI maps of the same slice as shown in Fig. 1. The lesion mask, marked by an arrow, was superimposed onto the FA, MD, AD, and RD maps to calculate median values for each lesion at every timepoint

The new lesions and corresponding contralateral/reference NAWM masks were drawn at the timepoint where the lesions appeared smallest and superimposed onto the individual MT/DTI parameter maps (Figs. 1 and 2) of all previous and following MRI scans. Lesions’ registration was performed and confirmed by an experienced MR physicist. In cases of inaccuracy, the latter was repeated and manually corrected.

The median across all lesional and corresponding NAWM voxel values was calculated for all maps (MTR, 4 qMT parameters, 4 DTI parameters). The relative intensity difference to the NAWM was calculated for every new lesion at each timepoint for each parameter. The mean and standard error of the relative intensity difference to the NAWM were plotted for each timepoint across all lesions. Paired-sample *t*-tests with Bonferroni correction served for comparison between pre-lesional and contralateral NAWM tissue at the timepoints before lesions’ detection on cMRI.

## Results

### EDSS

The mean disease duration (time between first symptoms and first MRI within the study) of all 19 patients was 11.7 years, SD = 8.2 years (range 2 months–29 years).

The patients’ median expanded disability status scale (EDSS) score remained quite stable during the first study year with 2.5 at the 1st MRI (month 0, IQR 2.0) and 7th MRI (month 12, IQR 1.5). There was a slight increase of the EDSS score at the end of the study (month 24, 8th MRI) with 3.0 (IQR 1.9).

EDSS scores were obtained on the same day of the respective MRI examination except for one patient at month 10 and for five patients at month 24 with EDSS scores assessed some days/weeks prior/later.

### New lesions

The total number of MS lesions markedly exceeded the amount of 500 at the baseline MRI (a precise lesion number cannot be provided as some patients showed partially largely confluent lesions).

Seven out of 19 patients developed a total of 21 new lesions (one to nine lesions per patient, all female, age range 25–54 years) (mean 37.7 years, SD = 10.8 years) with a mean disease duration of 8.6 years (SD = 3.7 years, range 2 months–16 years) and a median EDSS of 2 (IQR = 0.5, range 1–3.5) at the beginning, of 2.25 (IQR = 0.9, range 1–3.5) at month 12, and of 2 (IQR = 1.0, range 1.5–3.5) at month 24 of the study, respectively.

**Fig. 3:**
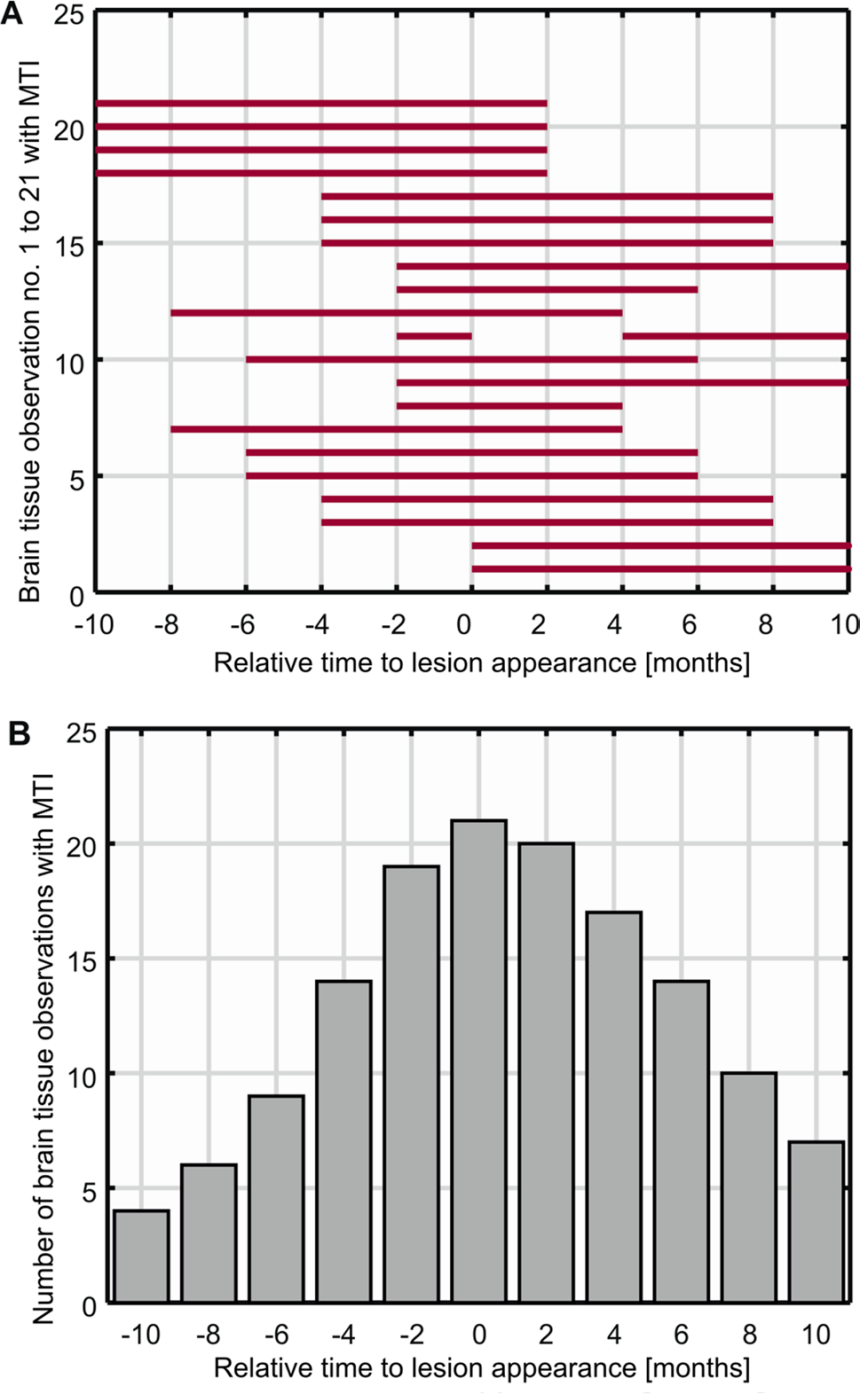
New MS lesions analyzed with MTI. Shown are the timepoints of brain tissue observations in the ROIs of the 21 newly appearing lesions detected in 19 MS patients. The observations analyzed with MTI (qMT and MTR) are numbered consecutively (1 to 21) and displayed as red bars. The interruptions of the bars represent missed MR examinations. The lesions are numbered from 1 to 21 and temporally centered on their appearance (month 0), so that 21 measured lesions are present at month 0. Because of the centering on month 0, less than 21 tissue observations are present at the other timepoints (**A**). Summing over the red bars in (**A**) results in the histogram of the number of brain tissue observations in the ROI of newly appearing lesions analyzed with qMT at each timepoint relative to the timepoint of their appearance (**B**)

Three of the 21 new lesions showed CE properties when detected. Figure 3A shows the timepoints of brain tissue observations in the ROIs of the newly appearing lesions as analyzed with qMT. They were centered up at the timepoint of their appearance. The corresponding histogram in Fig. 3B depicts the maximum number of brain tissue observations in the ROI of the newly appearing lesions analyzed with qMT at each timepoint relative to the timepoint of their appearance. The lesion volumes as segmented with qMT ranged from 12 to 201 mm^3^ (mean 71 ± 60 mm^3^).

**Fig. 4:**
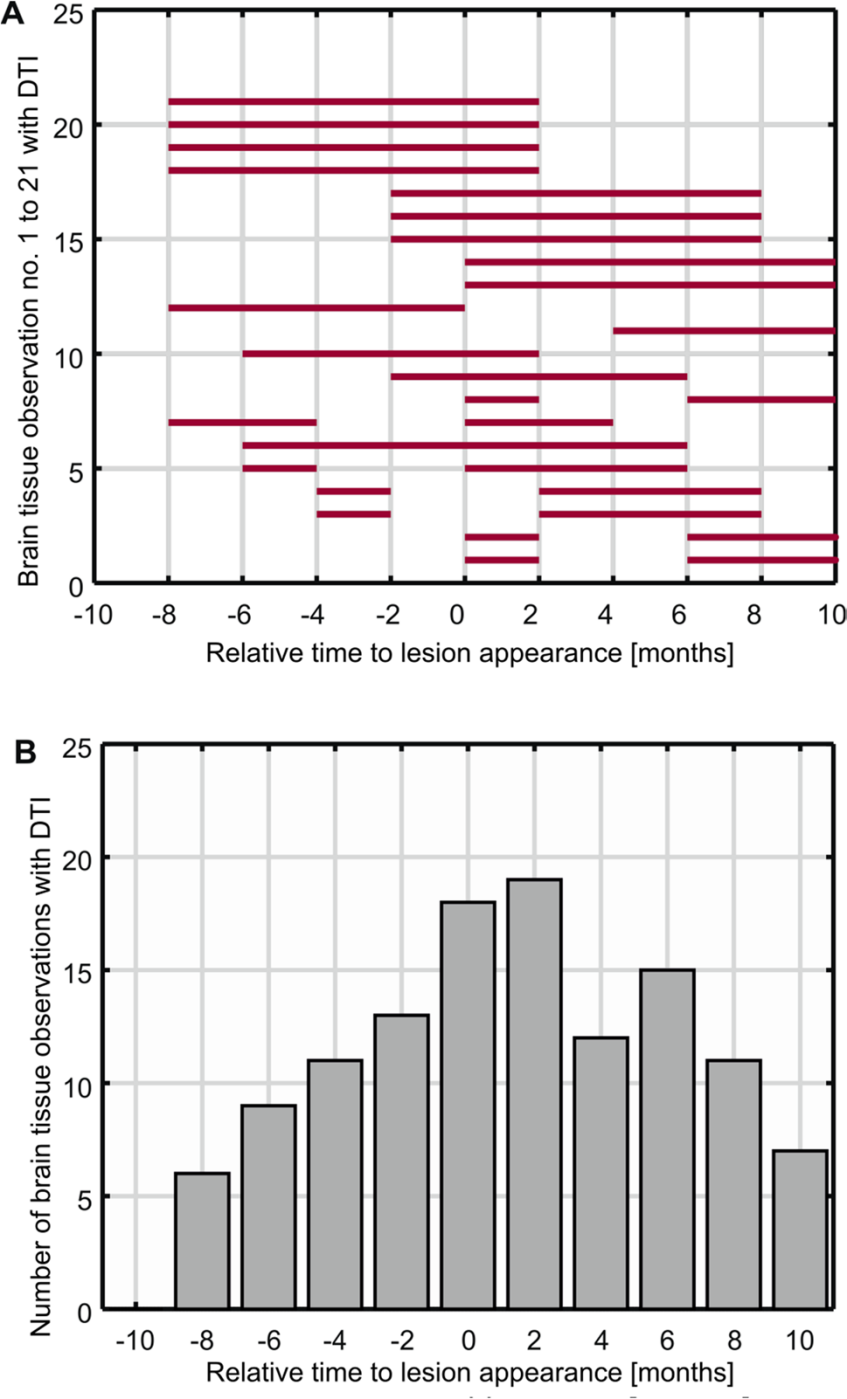
New MS lesions analyzed with DTI. Shown are the timepoints of brain tissue observations in the ROI of the 21 newly appearing lesions detected in 19 MS patients. The observations analyzed with DTI are numbered consecutively (1 to 21) and displayed as red bars. The interruptions of the bars represent missed MR examinations. The lesions were temporally centered on their appearance (month 0) (**A**). Because of the centering on month 0 and the fact that DTI was not always performed, less than 21 tissue observations are present at the other individual timepoints (**A**). Summing over the red bars in (**A**) results in the histogram of the number of brain tissue observations in the ROI of newly appearing lesions analyzed with DTI at each timepoint relative to the timepoint of their appearance (**B**)

DTI was analyzed in the same 21 new lesions. Correspondingly, Fig. 4A illustrates the timepoints of brain tissue observations in the ROIs of the newly appearing lesions as analyzed with DTI. They were centered up at the timepoint of their appearance. The corresponding histogram in Fig. 4B reflects the maximum number of brain tissue observations in the ROI of the newly appearing lesions analyzed with DTI at each timepoint relative to the timepoint of their appearance. The lesion volumes as segmented with DTI ranged from 21 to 262 mm^3^ (mean 100 ± 72 mm^3^).

### MTI and DTI before the lesions’ detection on conventional MRI

**Fig. 5:**
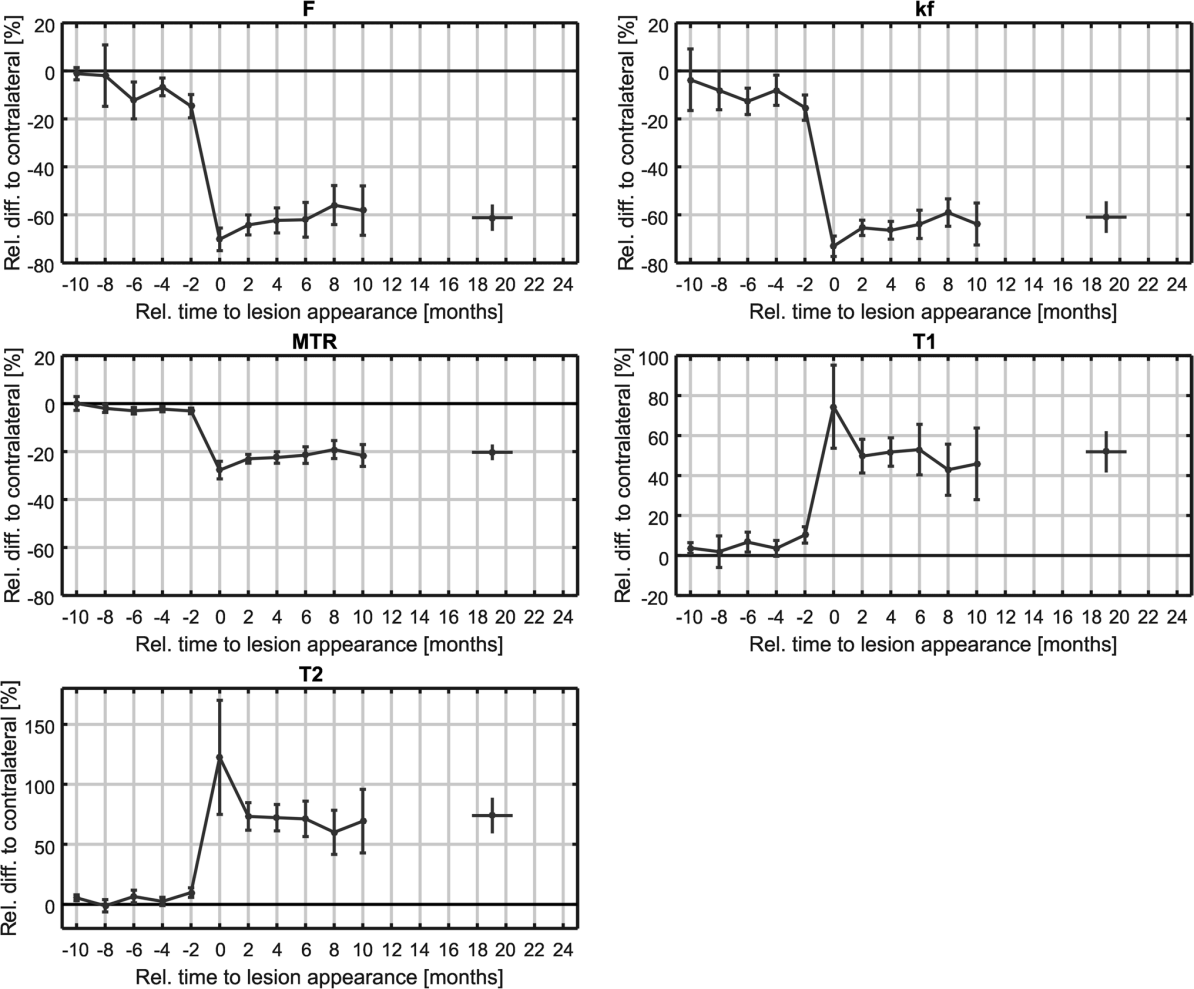
Temporal evolution of new MS lesions assessed by MTI parameters (F, kf, MTR, and relaxation times T1 and T2). The mean value and 95% confidence interval for the ROI of newly appearing lesions of 19 MS patients are shown. The last follow-up was 24 months after the first acquisition and has a temporal distribution resulting from the centering on lesion appearance

Ten to 4 months prior to lesions’ detection, the values for the differences (mean and 95% CI) for MT parameters relative to contralateral NAWM (representing 0%) are shown in Table 2 with reduced F/kf/MTR and increased T1/T2 values of lesions compared to contralateral WM. The MTR decrease of − 2.3% was already significant 4 months before lesions’ detection (*p* < 0.05), (Fig. 5; Tables 2 and 3).

**Table 2:** MTI parameters before, at the timepoint, and after lesions’ detection on conventional MRI

	10–4 months prior to lesions’ detection	2 months before lesions’ detection	Timepoint of lesions’ detection	2–8 months after lesions’ detection
F	− 1.2% (CI: − 3.7%, 1.4%) and − 12.3% (CI: − 20.0%, − 4.6%)	− 14.7% (CI: − 19.5%, − 9.9%) (*p* < 0.002) *	− 70.2% (CI: − 74.9%, − 65.5%) (*p* < 0.05)*	− 55.9% (CI: − 64.1%, − 47.8%)
kf	− 3.7% (CI: − 16.5%, 9.1%) and − 12.7% (CI: − 18.2%, − 7.2%)	− 15.3% (CI: − 20.6%, − 10.0%) (*p* < 0.002) *	− 73.1% (CI: − 77.3%, − 68.8%) (*p* < 0.05)*	− 59.1% (CI: − 64.8%, − 53.3%)
T1	+ 1.9% (CI: − 6.1%, 9.8%) and + 6.7 (CI: 1.7%, 11.7%)	+ 10.3% (CI: 6.2%, 14.4%) (*p* < 0.002) *	+ 74.5% (CI: 53.7%, 95.3%) (*p* < 0.05)*	+ 42.9% (CI: 30.1%, 55.7%)
T2	− 1.1% (CI: − 6.3%, 4.0%) and + 6.6% (CI: 1.3%, 11.9%)	+ 9.9% (CI: 5.9%, 13.9%) (*p* < 0.002) *	+ 122.5% (CI: 74.9%, 170.1%) (*p* < 0.05)*	+ 60% (CI: 41.6%, 78.3%)
MTR	+ 0.1% (CI: − 2.8%, 2.9%) and − 3.0% (CI: − 4.3%, − 1.6%), significant 4 months prior to lesions detection with − 2.3% (CI: − 3.4%, − 1.2%) (*p* < 0.05)*	− 3.0% (CI: − 4.2%, − 1.9%) (*p* < 0.002) *	− 27.7% (CI: − 31.4%, − 24.0%) (*p* < 0.05)*	− 19.1% (CI: − 22.9%, − 15.4%)

Shown are the differences or ranges of differences (mean and 95% CI) for the single MTI parameters (MTR and qMT) relative to the contralateral NAWM (representing 0%). Significances are marked with a star

**Table 3:** *t*-tests comparing MTI parameters (qMT and MTR) of pre-lesional tissue and contralateral tissue across patients at each individual timepoint before lesions’ appearance

Rel. time	Number of MTI brain tissue observations	F	kf	T1	T2	MTR
[months]	No.	*p*-value	*p*-value	*p*-value	*p*-value	*p*-value
− 10	4	0.435	0.567	0.073	0.022	0.986
− 8	6	0.763	0.105	0.652	0.712	0.065
− 6	9	0.013	0.003	0.029	0.037	0.003
− 4	14	0.04	0.022	0.101	0.176	0.001*
− 2	19	2.33E − 05*	4.06E − 05*	6.80E − 05*	5.40E − 05*	7.03E − 05*

The significance level for the whole test is *α* = 0.05. The Bonferroni-corrected significance level for each of the 25 hypotheses is α* = 0.002 and significant *p*-values are marked with a star

Two months before lesions’ detection, the relative intensity differences of the “pre-lesional” tissue were more pronounced with significances (*p* < 0.002) for all MT values with F/kf/MTR showing reduced and T1/T2 showing increased values compared to contralateral NAWM (Fig. 5; Tables 2 and 3).

**Fig. 6:**
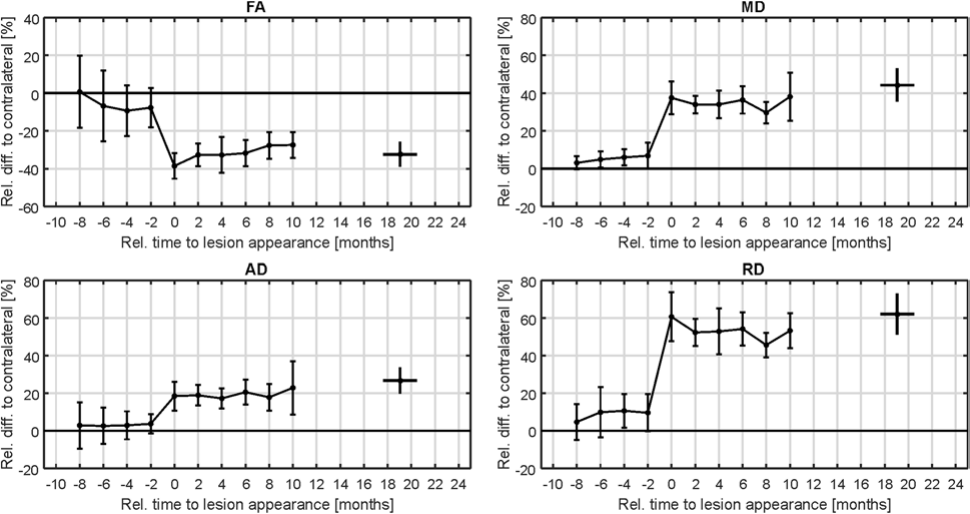
Temporal evolution of new MS lesions assessed by DTI parameters (FA, MD, AD, and RD). The mean value and 95% confidence interval for the newly appearing lesions of 19 MS patients are shown. The last follow-up was 24 months after the first acquisition and has a temporal distribution resulting from the centering on lesion appearance

Ten to 2 months before lesions’ detection, DTI parameters did not show any significant changes (*p* > 0.05) in the “pre-lesional” tissue compared to contralateral NAWM, with a decreased FA value and increased MD/AD/RD values (Fig. 6; Tables 4 and 5).

**Table 4:** DTI parameters before, at the timepoint, and after lesions’ detection on conventional MRI

	10–2 months prior to lesions’ detection	Timepoint of lesions’ detection	2–8 months after lesions’ detection
FA	− 9.3% (CI: − 22.8%, 4.1%)	− 38.5% (CI: − 45.3%, − 31.8%) (*p* < 0.05)*	− 27.7% (CI: − 34.7%, − 20.6%)
MD	6.8% (CI: 0.0%, 13.7%)	+ 37.5% (CI: 28.8%, 46.1%) (*p* < 0.05)*	+ 29.6% (CI: 24.0%, 35.3%)
AD	3.6% (CI: − 1.4%, 8.7%)	18.4% (CI: 10.7%, 26.1%) (*p* < 0.05)*	+ 17.8% (CI: 10.6%, 24.9%)
RD	10.6% (CI: 1.7%, 19.5%)	+ 60.6% (CI: 47.6%, 73.5%) (*p* < 0.05)*	+ 45.6% (CI: 39.0%, 52.1%),

Shown are the differences (mean and 95% CI) for the single DTI parameters relative to the contralateral NAWM (representing 0%). Significances are marked with a star

**Table 5:** *t*-tests comparing DTI parameters of pre-lesional tissue and contralateral tissue across patients at each individual timepoint before lesions’ appearance

Rel. time	Number of DTI brain tissue observations	FA	MD	AD	RD
[months]	No.	*p*-value	*p*-value	*p*-value	*p*-value
− 8	6	0.566	0.279	0.434	0.638
− 6	9	0.573	0.326	0.206	0.904
− 4	11	0.508	0.070	0.081	0.176
− 2	13	0.156	0.318	0.393	0.300

The significance level for the whole test is *α* = 0.05. The Bonferroni-corrected significance level for each of the 16 hypotheses is α* = 0.003 and significant *p*-values are marked with a star

### MTI and DTI at the time of the lesions’ detection on conventional MRI

At the timepoint of the lesions’ detection, average differences of all individual MT parameters and also of all DTI parameters (mean and 95% CI) between lesions and contralateral NAWM were highly significant (*p* < 0.05) with relative differences for MT parameters as given in Table 2 and Fig. 5. As expected, F/kf/MTR values were markedly reduced and T1/T2 values were markedly increased. For DTI parameters, MS lesions showed significantly reduced FA and significantly increased MD/AD/RD values (*p* < 0.05) (Fig. 6; Table 4).

### MTI and DTI after the lesions’ appearance on conventional MRI

Two to 8 months after lesions’ detection, MTI parameters of MS lesions showed a tendency to normalization compared to contralateral NAWM-ROIs as shown in Table 2. Ten months after lesions’ detection, MTI parameters showed a slightly reversed tendency with quite stable parameters at 24 months (Fig. 5).

For DTI, a slight normalization tendency up to 8 months after lesions’ detection was observed for FA/RD/MD whereas AD stayed largely unchanged (Table 4). The assessed DTI parameters became slightly worse for MD/RD/AD 10 months and for all four DTI parameters 24 months after lesions’ appearance. No MTI/DTI parameter reached its respective value prior to lesions’ detection (Fig. 6).

## Discussion

Various methods, including MTR, normalized T2 intensity, DWI, and MR spectroscopy, have shown differences between the pre-lesional NAWM and the non-pre-lesional NAWM in MS patients [15, 23–25].

Studies on MTI changes in MS lesions prior to their identification on cMRI report no relevant changes [9, 26] to statistically significant changes ranging from 3 months to 2 years before their appearance [10, 11, 27]. Fazekas et al observed a significant reduction in MTR/kf 4 months and a significant increase in T1 3 months, and Goodkin et al described MTR/T2 changes several months before lesions’ detection [27, 28].

In our study, all four qMT parameters were significantly altered in the pre-lesional NAWM 2 months and MTR 4 months before lesions’ detection.

These inconsistencies may be attributed to technical differences [29]. Previous studies mainly relied on MTR assessment only or on qMT-based gradient-echo sequences with rather low resolution, low SNR, and long TA [9–11, 27]. This study is the first one tracking the evolution of MS lesions with MTR and four qMT parameters retro- and prospectively using fast 3D-bSSFP.

Our study was quite consistent with two previous studies describing an MTR reduction 3 to 4 months before lesions’ appearance [10, 28] and with two qMT studies using lower resolution [27, 28].

Only three new lesions showed CE properties when detected, i.e., most new lesions had already lost their CE properties during the preceding 2-month imaging interval.

Greatest MTR/qMT changes were observed at the timepoint of the lesions’ detection [6, 11, 17, 30]. The T1 increase has been attributed to edema and reported to attenuate MTR changes [30]. Ropele et al [12], however, found similar MTR values in edematous and T1w isointense tissue, and Levesque et al [6] reported that in chronic black holes the T1 increase correlated stronger with a decline in F than with edema, altogether illustrating the textural complexity of MS lesions [31, 32].

From 2 to 8 months after lesions’ detection, a recovery tendency could be observed for all MT parameters, more pronounced for qMT, and by + 12 months, the MT parameter values were still distant from their pre-lesional baseline.

A partial MTR recovery has also been observed in new and repeat lesions [33], and Levesque et al observed a partial recovery for qMT parameters but not for myelin water estimates in CE lesions [6]. In MS patients, qMT revealed a better correlation of NAWM, gray matter (GM), and MS lesions with clinical scores than MTR [34]. These observations, together with findings from previous studies using bSSFP-based MTI [17, 35], suggest the superiority of qMT over MTR, not excluding an ongoing recovery from previous inflammation years after lesions’ detection [6].

The interpretation of reduced MT in WM demyelination has been shown in animal models [36–38] demonstrating an increased distance between axons due to a dilution of the axonal and myelin concentration. In unfixed brain slices of MS patients, the MT pool size fractions strongly correlated with myelin staining and axonal concentration [37]. The implied high MTI sensitivity for myelin might explain the earlier detection of structural changes with MTI compared to cMRI. More qMT studies are required for a better translation of these cellular processes into the level of MRI [38].

Multiple studies with DWI/DTI [1–4] in MS largely agree on the DWI/DTI parameter changes [39, 40].

Studies assessing MS lesions before their detection are also scarce for DWI/DTI showing inconsistencies [4, 5], possibly due to methodical differences.

An FA reduction and MD/AD/RD increase were observed prior to lesions’ detection, without significance. In consensus with previous studies, significant DTI parameter changes were observed at the timepoint of lesions’ identification, and a slightly recidivous tendency was observed for three parameters 10 months and for all four assessed parameters 24 months after lesions’ detection. The partially expected divergent results between MTI and DTI can be explained by the inherently different techniques themselves and the complex pathophysiological process behind MS lesions. Nonetheless, standard DTI may be less sensitive for structural changes in MS lesions compared to MTI.

More advanced techniques derived on diffusion, e.g., assessment of the full tensor itself, myelin water fraction, and myelin water imaging, or T^★^2 and quantitative susceptibility mapping are promising for providing pathophysiological information from MS lesions [6, 7, 32, 41–45]. With diffusion basis spectrum imaging consistent results with histological findings regarding the differentiation and quantification of inflammation, demyelination and axonal injury/loss in MS have been shown [46]. However, the few publications available on these techniques hamper firm conclusions on which technique might be the most promising one in the clinical setting [32].

One explanation for the MTI/DTI inconsistencies in MS between studies might be, apart from differences in imaging parameters and techniques, the a priori altered NAWM hampering a veritable differentiation of pre-lesional NAWM from non-pre-lesional NAWM and/or DWM [6, 10–13, 32]. The NAWM/DWM probably differs between patients, e.g., depending on the stage of the disease. Our study shows a rather large disease duration, probably partially affecting the results. However, the aim of this study was not to investigate the NAWM/DWM per se. Being aware that the NAWM in MS patients is not “normal,” it nevertheless served as a reference as the aim of this study was to longitudinally assess new MS lesions based on a standardized reference.

Further limitations include the slightly accentuated data dispersion of qMT parameters, even if MT-bSSFP has proven to be a stable technique. Its stability could be strengthened by longer TAs. The 2-month time interval between examinations might affect data homogeneity, too.

The unexpectedly unfavorably rather low new lesion number could not be influenced by its natural course. This prevented lesions’ subdivision into subtypes based on previous observations showing divergent dynamics of different lesion types or the determination of future lesions relying on different pre-lesional measures [47–49].

The patients were not in the same treatment (pre)conditions. To include only patients with the same or without treatment is an unswayable limitation of the study.

Fast whole-brain bSSFP-based MTI (< 10 min) can be additionally acquired in routine MRI assessment. Contrary to DTI, with MTI, MS lesions could be detected 2 to 4 months earlier than with cMRI. QMT changes were more pronounced than MTR changes after lesions’ appearance, indicating its superiority regarding assessment of potential reparative processes.

To what extent the “earlier” detection of MS lesions with the presented MTI technique could implicate an earlier treatment initiation and better follow-up/treatment assessment [30] remains to be assessed by more studies in this field.

## Abbreviations

AD: Axial diffusivity
bSSFP: Balanced steady-state free precession
CE: Contrast-enhancing
CI: Confidence interval
cMRI: Conventional MRI
DMT: Disease-modifying therapy
DTI: Diffusion tensor imaging
DWI: Diffusion-weighted imaging
DWM: Dirty white matter
EDSS: Expanded disability status scale
F: Pool size ratio
FA: Fractional anisotropy
FLAIR: Fluid-attenuated inversion recovery
IQR: Interquartile range
kf: Exchange rate
MD: Mean diffusivity
MPRAGE: Magnetization-prepared rapid gradient-echo
MS: Multiple sclerosis
MT: Magnetization transfer
MTI: Magnetization transfer imaging
MTR: Magnetization transfer ratio
NAWM: Normal-appearing white matter
NWM: Normal white matter
PDw: Proton density–weighted
QMT: Quantitative magnetization transfer
RD: Radial diffusivity
RF: Radiofrequency
RRMS: Relapsing-remitting MS
SD: Standard deviation
SPMS: Secondary progressive MS
T1/T2: Relaxation parameters
TA: Acquisition time

## Declarations

### Guarantor

The scientific guarantor of this publication is Prof. Dr. Oliver Bieri.

### Statistics and biometry

Two of the authors (Dr. Monika Gloor and Prof. Dr. Oliver Bieri) have significant statistical expertise.

### Study subjects or cohorts overlap

The subjects or cohorts have not been previously reported anywhere.

### Methodology

• prospective

• observational

• performed at one institution
